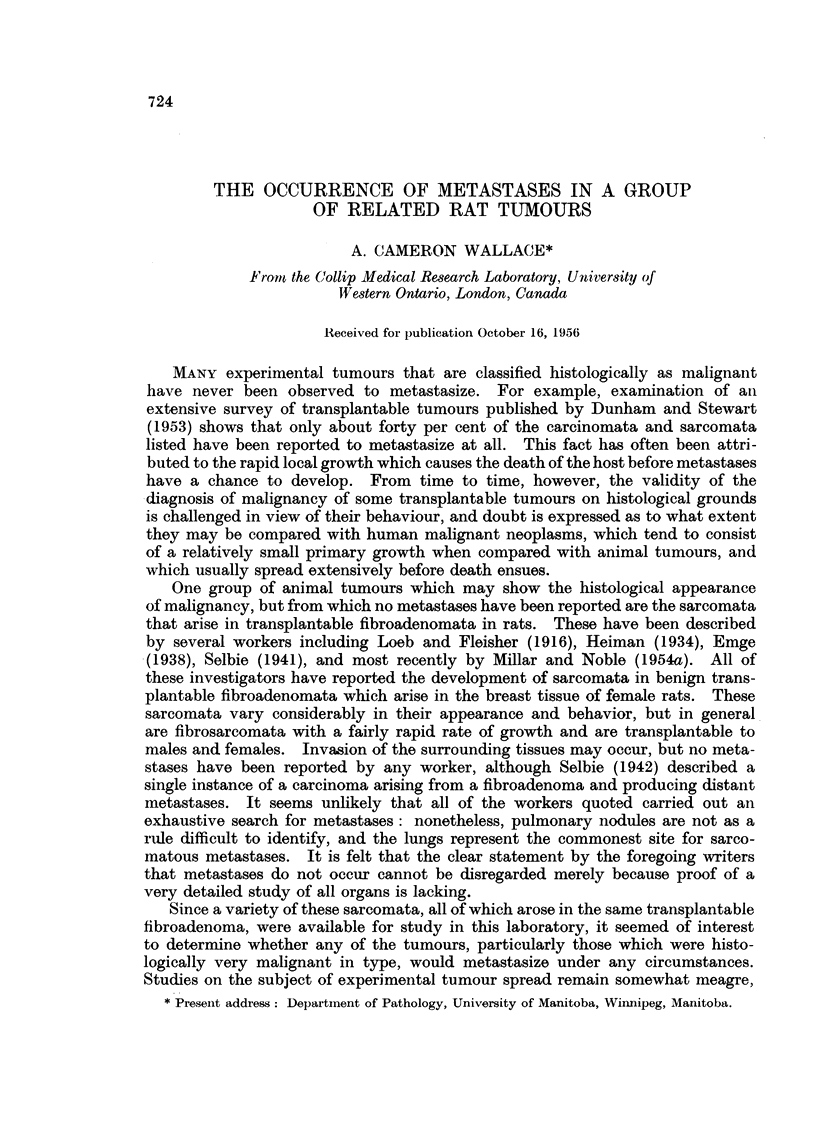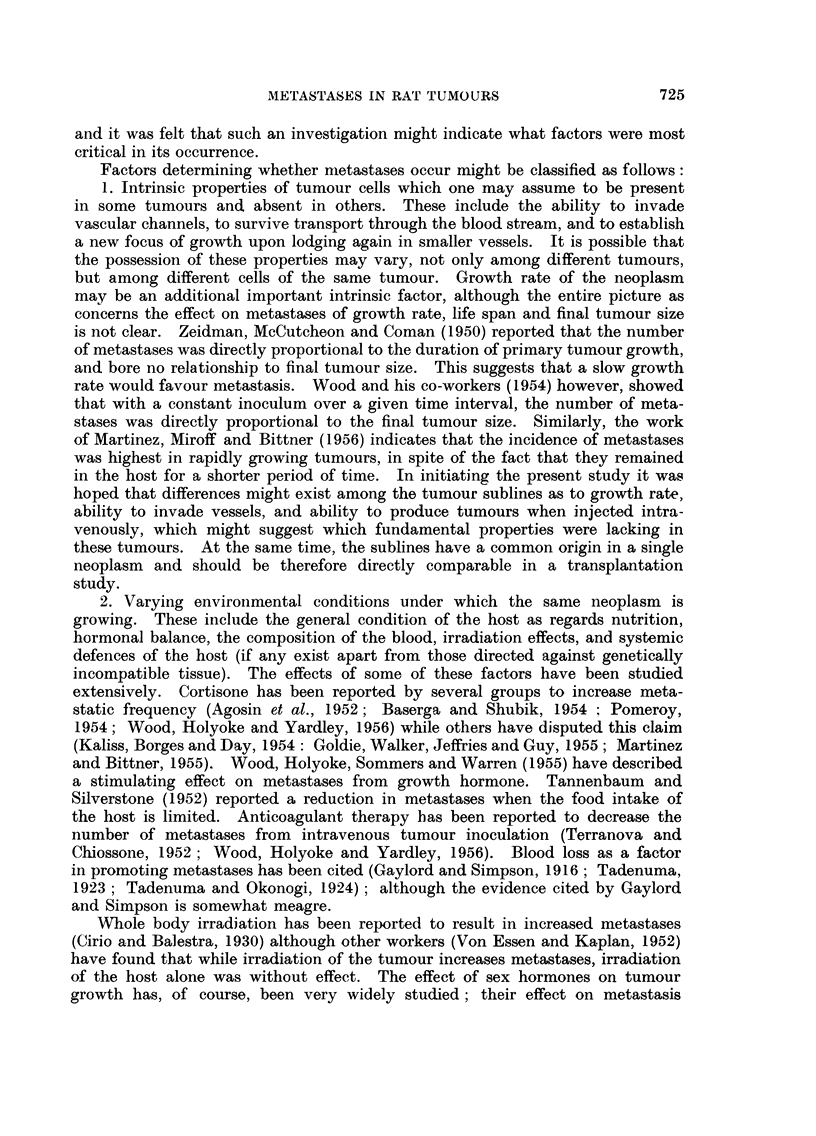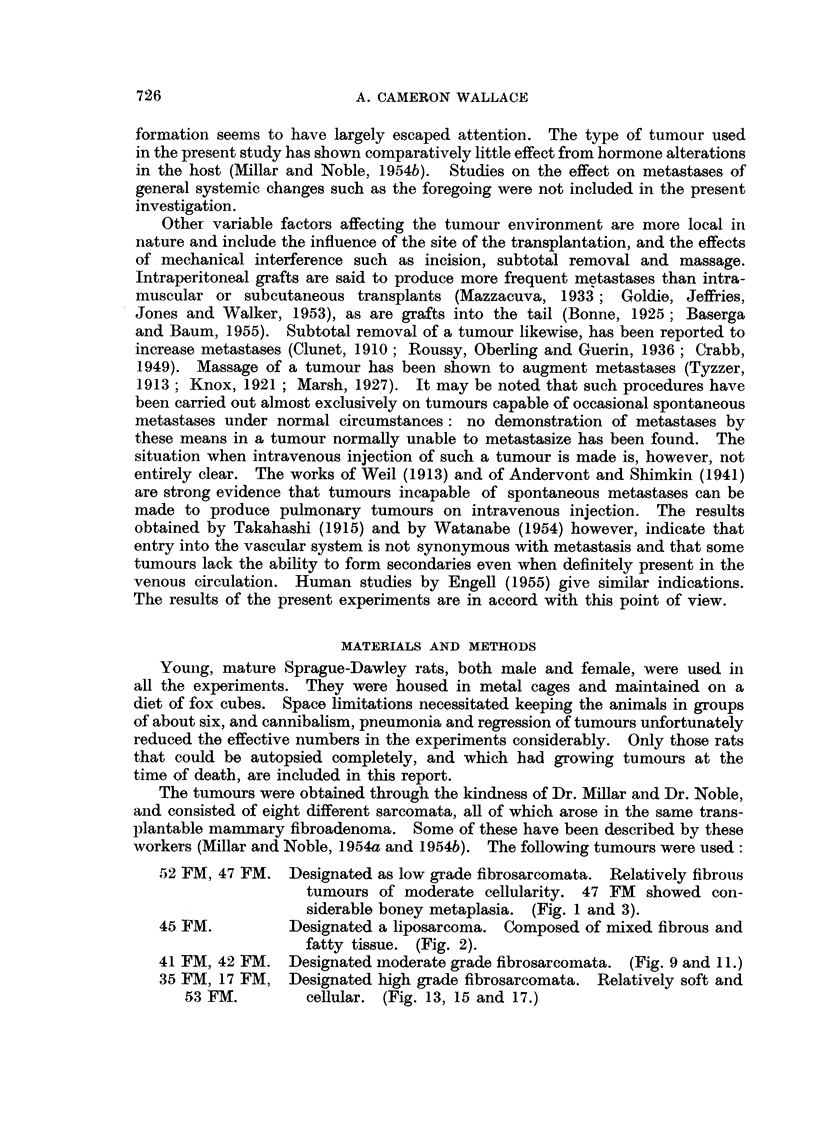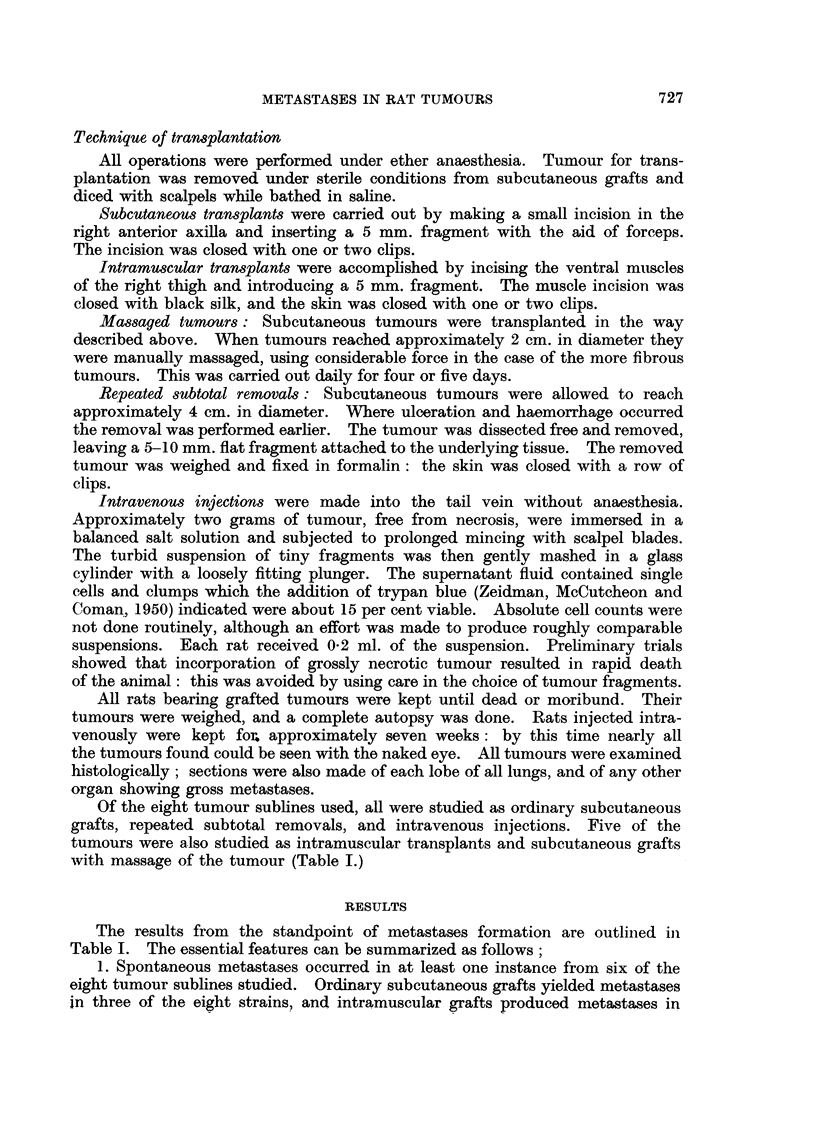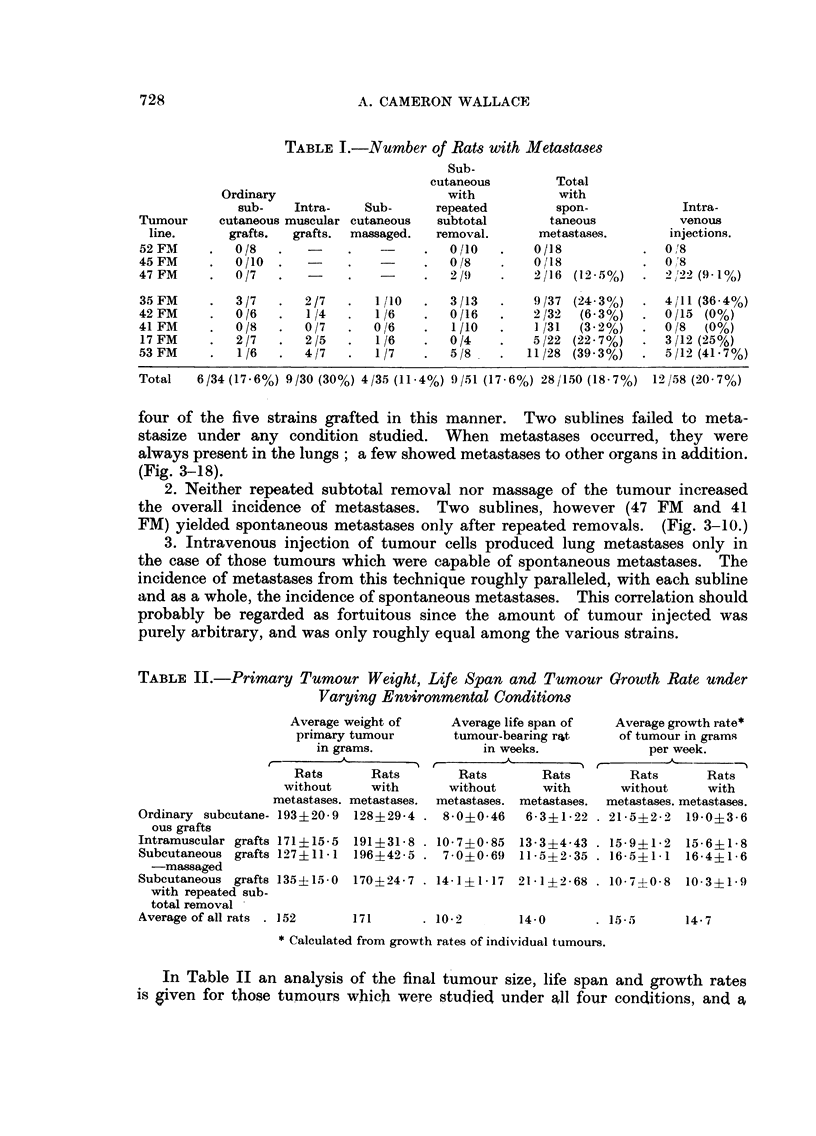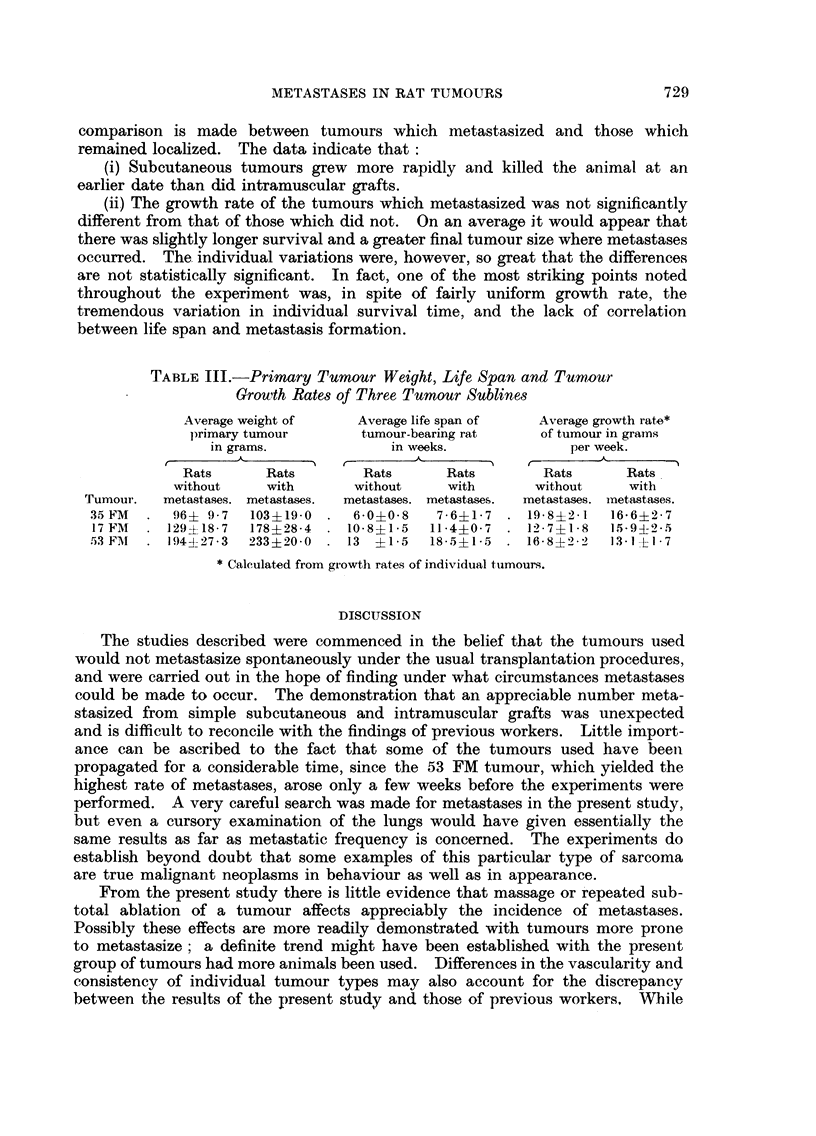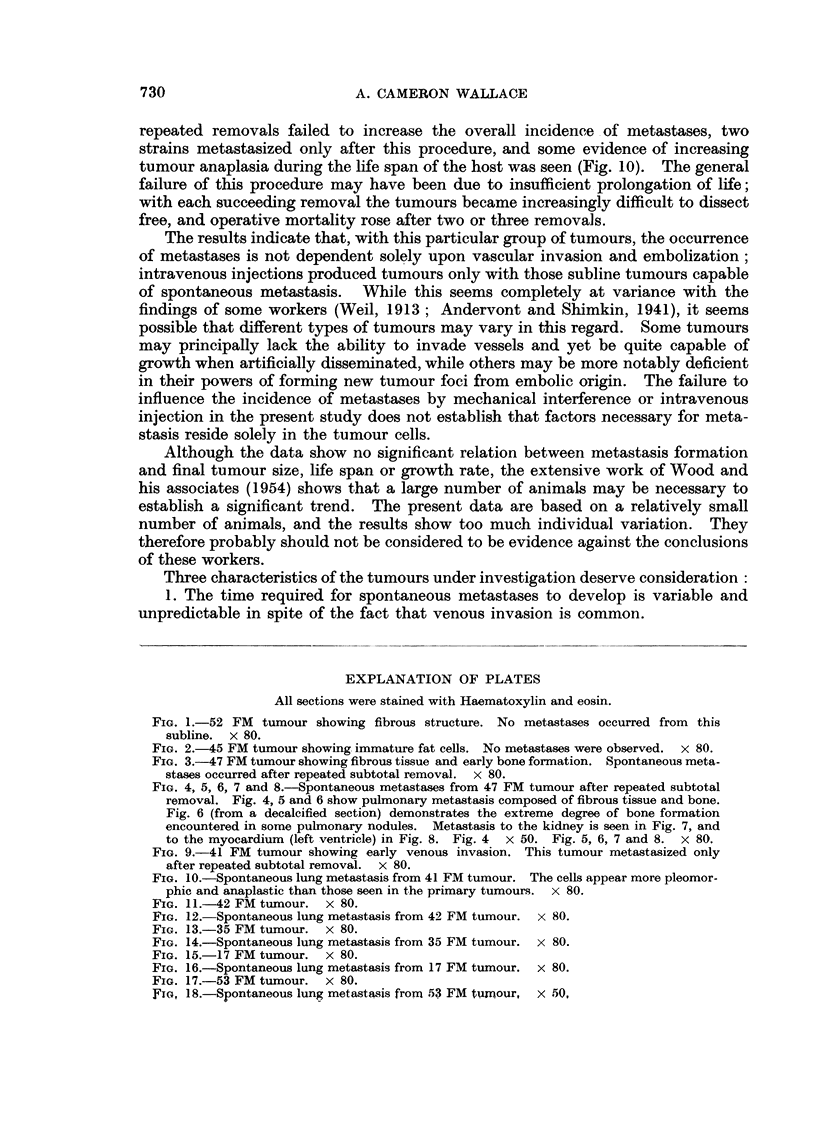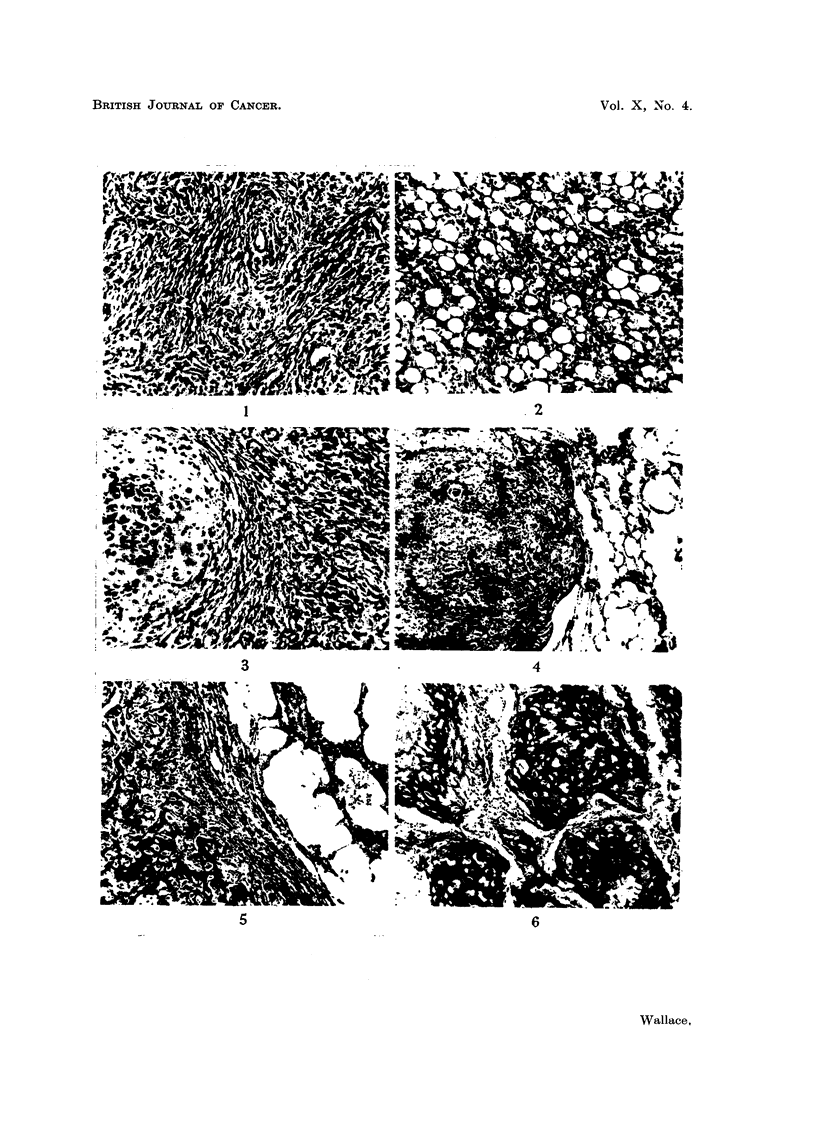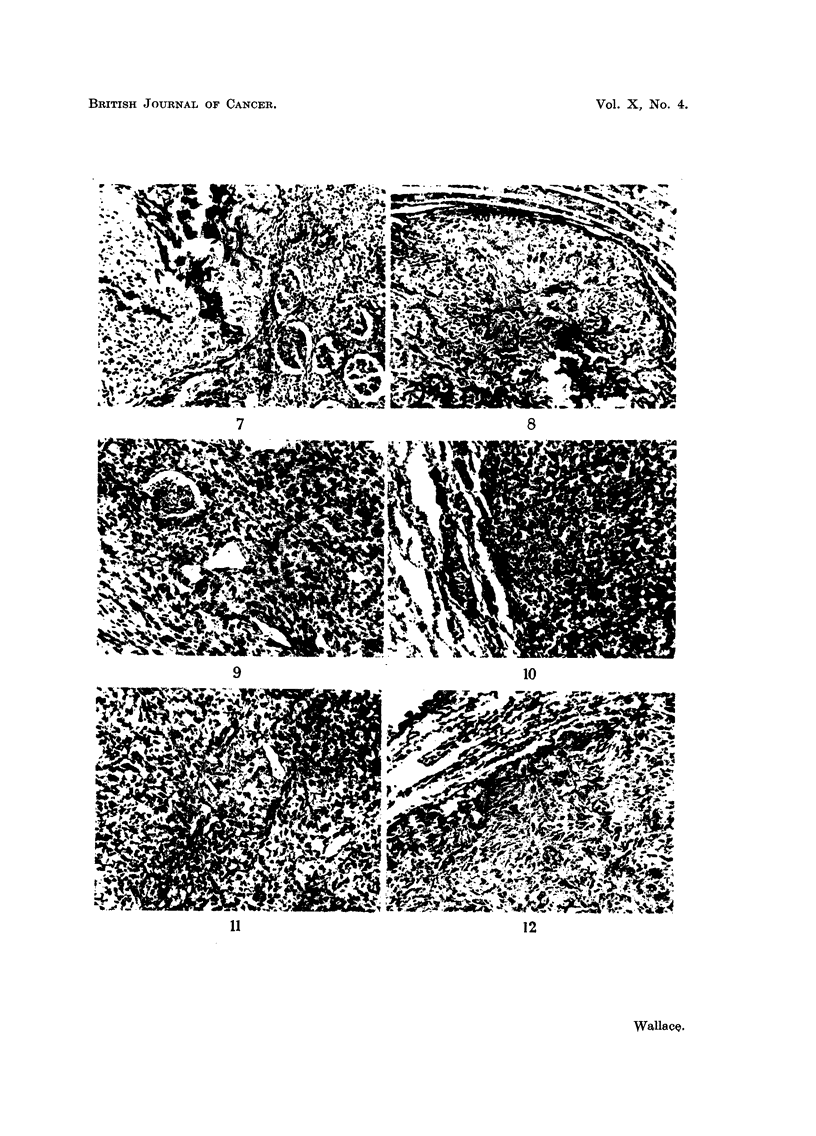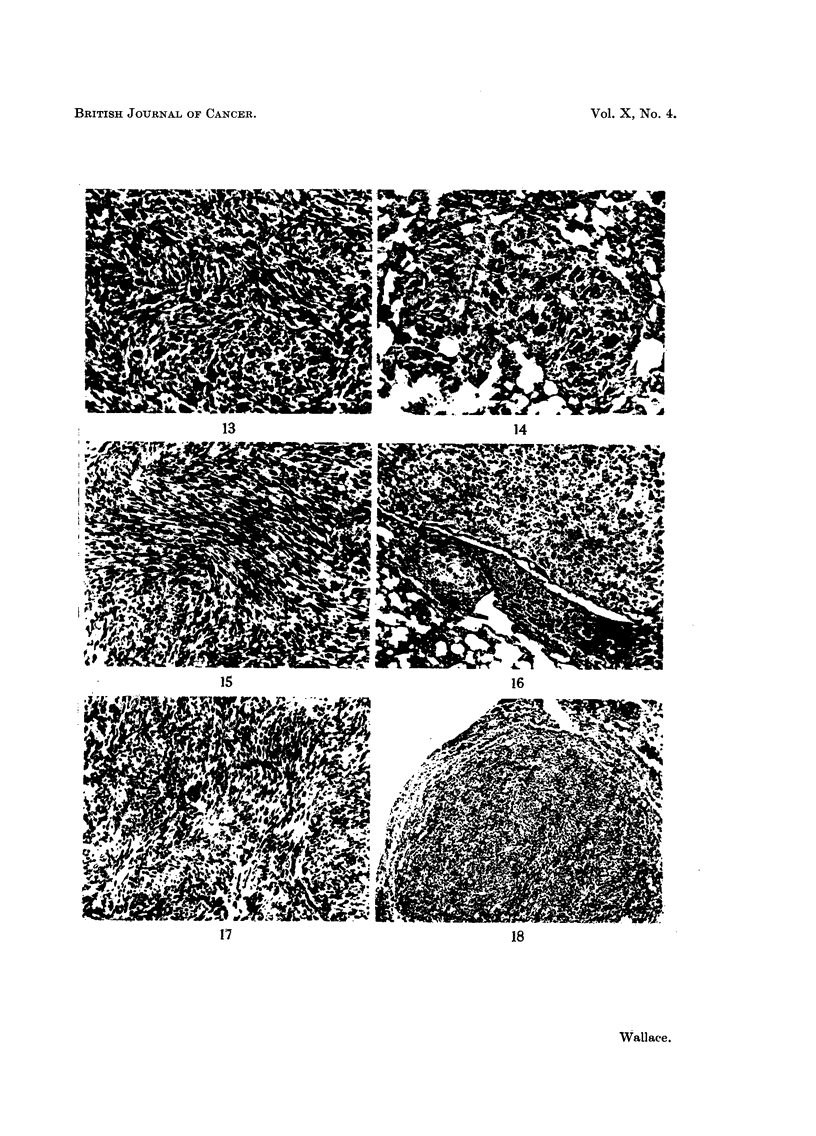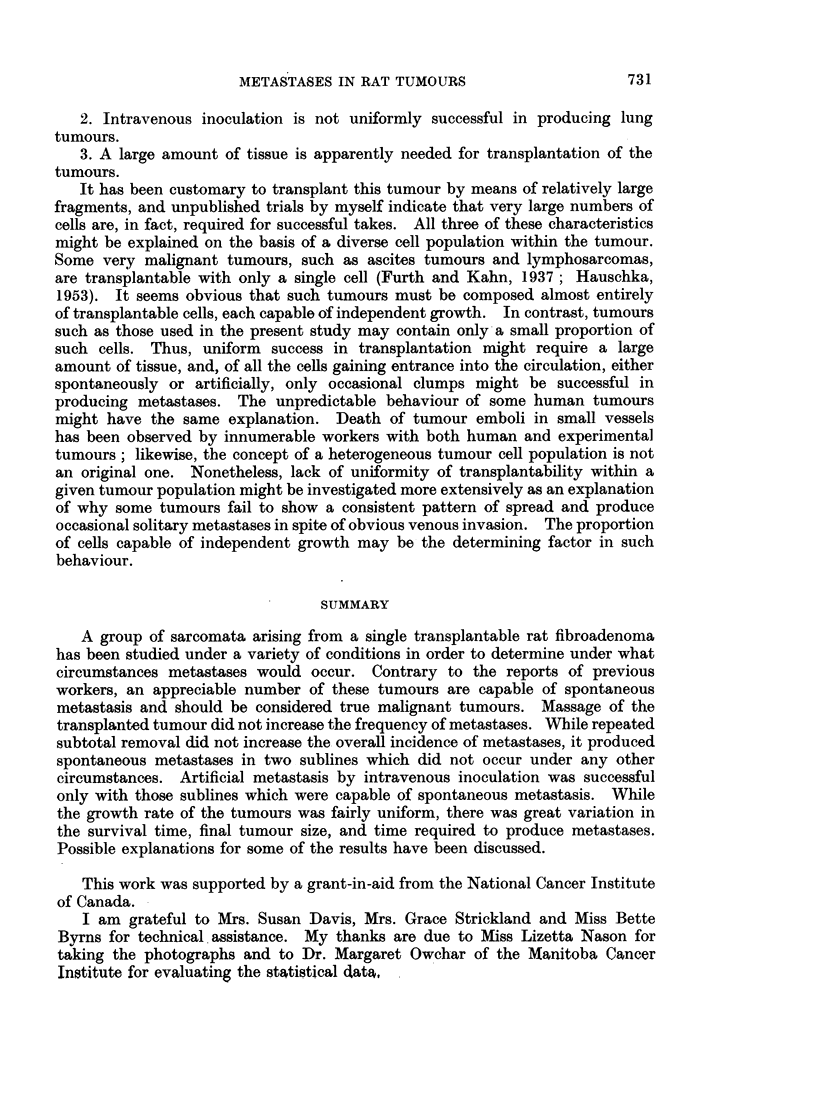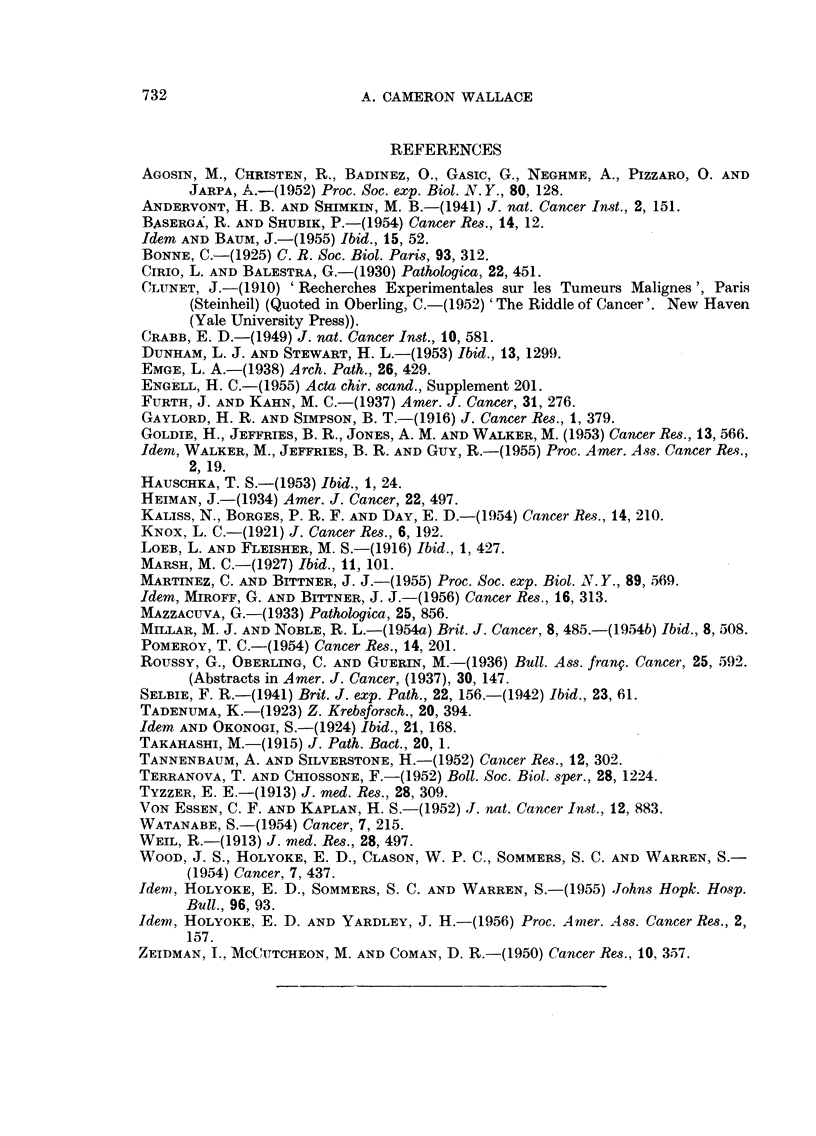# The Occurrence of Metastases in a Group of Related Rat Tumours

**DOI:** 10.1038/bjc.1956.88

**Published:** 1956-12

**Authors:** A. Cameron Wallace

## Abstract

**Images:**


### THE OCCURRENCE OF METASTASES IN A GROUP OF RELATED RAT TUMOURS

A. CAMERON WALLACE*

*From the Collip Medical Research Laboratory, University of Western Ontario, London, Canada*

Received for publication October 16, 1956

MANY experimental tumours that are classified histologically as malignant have never been observed to metastasize. For example, examination of an extensive survey of transplantable tumours published by Dunham and Stewart (1953) shows that only about forty per cent of the carcinomata and sarcomata listed have been reported to metastasize at all. This fact has often been attributed to the rapid local growth which causes the death of the host before metastases have a chance to develop. From time to time, however, the validity of the diagnosis of malignancy of some transplantable tumours on histological grounds is challenged in view of their behaviour, and doubt is expressed as to what extent they may be compared with human malignant neoplasms, which tend to consist of a relatively small primary growth when compared with animal tumours, and which usually spread extensively before death ensues.

One group of animal tumours which may show the histological appearance of malignancy, but from which no metastases have been reported are the sarcomata that arise in transplantable fibroadenomata in rats. These have been described by several workers including Loeb and Fleisher (1916), Heiman (1934), Emge (1938), Selbie (1941), and most recently by Millar and Noble (1954*a*). All of these investigators have reported the development of sarcomata in benign transplantable fibroadenomata which arise in the breast tissue of female rats. These sarcomata vary considerably in their appearance and behavior, but in general are fibrosarcomata with a fairly rapid rate of growth and are transplantable to males and females. Invasion of the surrounding tissues may occur, but no metastases have been reported by any worker, although Selbie (1942) described a single instance of a carcinoma arising from a fibroadenoma and producing distant metastases. It seems unlikely that all of the workers quoted carried out an exhaustive search for metastases : nonetheless, pulmonary nodules are not as a rule difficult to identify, and the lungs represent the commonest site for sarcomatous metastases. It is felt that the clear statement by the foregoing writers that metastases do not occur cannot be disregarded merely because proof of a very detailed study of all organs is lacking.

Since a variety of these sarcomata, all of which arose in the same transplantable fibroadenoma, were available for study in this laboratory, it seemed of interest to determine whether any of the tumours, particularly those which were histologically very malignant in type, would metastasize under any circumstances. Studies on the subject of experimental tumour spread remain somewhat meagre,

* Present address : Department of Pathology, University of Manitoba, Winnipeg, Manitoba.

and it was felt that such an investigation might indicate what factors were most critical in its occurrence.

Factors determining whether metastases occur might be classified as follows:

1. Intrinsic properties of tumour cells which one may assume to be present in some tumours and absent in others. These include the ability to invade vascular channels, to survive transport through the blood stream, and to establish a new focus of growth upon lodging again in smaller vessels. It is possible that the possession of these properties may vary, not only among different tumours, but among different cells of the same tumour. Growth rate of the neoplasm may be an additional important intrinsic factor, although the entire picture as concerns the effect on metastases of growth rate, life span and final tumour size is not clear. Zeidman, McCutcheon and Coman (1950) reported that the number of metastases was directly proportional to the duration of primary tumour growth, and bore no relationship to final tumour size. This suggests that a slow growth rate would favour metastasis. Wood and his co-workers (1954) however, showed that with a constant inoculum over a given time interval, the number of metastases was directly proportional to the final tumour size. Similarly, the work of Martinez, Miroff and Bittner (1956) indicates that the incidence of metastases was highest in rapidly growing tumours, in spite of the fact that they remained in the host for a shorter period of time. In initiating the present study it was hoped that differences might exist among the tumour sublines as to growth rate, ability to invade vessels, and ability to produce tumours when injected intravenously, which might suggest which fundamental properties were lacking in these tumours. At the same time, the sublines have a common origin in a single neoplasm and should be therefore directly comparable in a transplantation study.

2. Varying environmental conditions under which the same neoplasm is growing. These include the general condition of the host as regards nutrition, hormonal balance, the composition of the blood, irradiation effects, and systemic defences of the host (if any exist apart from those directed against genetically incompatible tissue). The effects of some of these factors have been studied extensively. Cortisone has been reported by several groups to increase metastatic frequency (Agosin *et al.*, 1952; Baserga and Shubik, 1954 ; Pomeroy, 1954; Wood, Holyoke and Yardley, 1956) while others have disputed this claim (Kaliss, Borges and Day, 1954 : Goldie, Walker, Jeffries and Guy, 1955 ; Martinez and Bittner, 1955). Wood, Holyoke, Sommers and Warren (1955) have described a stimulating effect on metastases from growth hormone. Tannenbaum and Silverstone (1952) reported a reduction in metastases when the food intake of the host is limited. Anticoagulant therapy has been reported to decrease the number of metastases from intravenous tumour inoculation (Terranova and Chiossone, 1952 ; Wood, Holyoke and Yardley, 1956). Blood loss as a factor in promoting metastases has been cited (Gaylord and Simpson, 1916 ; Tadenuma, 1923 ; Tadenuma and Okonogi, 1924) ; although the evidence cited by Gaylord and Simpson is somewhat meagre.

Whole body irradiation has been reported to result in increased metastases (Cirio and Balestra, 1930) although other workers (Von Essen and Kaplan, 1952) have found that while irradiation of the tumour increases metastases, irradiation of the host alone was without effect. The effect of sex hormones on tumour growth has, of course, been very widely studied ; their effect on metastasis formation seems to have largely escaped attention. The type of tumour used in the present study has shown comparatively little effect from hormone alterations in the host (Millar and Noble, 1954*b*). Studies on the effect on metastases of general systemic changes such as the foregoing were not included in the present investigation.

Other variable factors affecting the tumour environment are more local in nature and include the influence of the site of the transplantation, and the effects of mechanical interference such as incision, subtotal removal and massage. Intraperitoneal grafts are said to produce more frequent metastases than intramuscular or subcutaneous transplants (Mazzacuva, 1933; Goldie, Jeffries, Jones and Walker, 1953), as are grafts into the tail (Bonne, 1925; Baserga and Baum, 1955). Subtotal removal of a tumour likewise, has been reported to increase metastases (Clunet, 1910; Roussy, Oberling and Guerin, 1936; Crabb, 1949). Massage of a tumour has been shown to augment metastases (Tyzzer, 1913; Knox, 1921; Marsh, 1927). It may be noted that such procedures have been carried out almost exclusively on tumours capable of occasional spontaneous metastases under normal circumstances: no demonstration of metastases by these means in a tumour normally unable to metastasize has been found. The situation when intravenous injection of such a tumour is made is, however, not entirely clear. The works of Weil (1913) and of Andervont and Shimkin (1941) are strong evidence that tumours incapable of spontaneous metastases can be made to produce pulmonary tumours on intravenous injection. The results obtained by Takahashi (1915) and by Watanabe (1954) however, indicate that entry into the vascular system is not synonymous with metastasis and that some tumours lack the ability to form secondaries even when definitely present in the venous circulation. Human studies by Engell (1955) give similar indications. The results of the present experiments are in accord with this point of view.

### MATERIALS AND METHODS

Young, mature Sprague-Dawley rats, both male and female, were used in all the experiments. They were housed in metal cages and maintained on a diet of fox cubes. Space limitations necessitated keeping the animals in groups of about six, and cannibalism, pneumonia and regression of tumours unfortunately reduced the effective numbers in the experiments considerably. Only those rats that could be autopsied completely, and which had growing tumours at the time of death, are included in this report.

The tumours were obtained through the kindness of Dr. Millar and Dr. Noble, and consisted of eight different sarcomata, all of which arose in the same transplantable mammary fibroadenoma. Some of these have been described by these workers (Millar and Noble, 1954*a* and 1954*b*). The following tumours were used:

52 FM, 47 FM. Designated as low grade fibrosarcomata. Relatively fibrous tumours of moderate cellularity. 47 FM showed considerable boney metaplasia. (Fig. 1 and 3).

45 FM. Designated a liposarcoma. Composed of mixed fibrous and fatty tissue. (Fig. 2).

41 FM, 42 FM. Designated moderate grade fibrosarcomata. (Fig. 9 and 11.)

35 FM, 17 FM, 53 FM. Designated high grade fibrosarcomata. Relatively soft and cellular. (Fig. 13, 15 and 17.)

*Technique of transplantation*

All operations were performed under ether anaesthesia. Tumour for transplantation was removed under sterile conditions from subcutaneous grafts and diced with scalpels while bathed in saline.

*Subcutaneous transplants* were carried out by making a small incision in the right anterior axilla and inserting a 5 mm. fragment with the aid of forceps. The incision was closed with one or two clips.

*Intramuscular transplants* were accomplished by incising the ventral muscles of the right thigh and introducing a 5 mm. fragment. The muscle incision was closed with black silk, and the skin was closed with one or two clips.

*Massaged tumours :* Subcutaneous tumours were transplanted in the way described above. When tumours reached approximately 2 cm. in diameter they were manually massaged, using considerable force in the case of the more fibrous tumours. This was carried out daily for four or five days.

*Repeated subtotal removals :* Subcutaneous tumours were allowed to reach approximately 4 cm. in diameter. Where ulceration and haemorrhage occurred the removal was performed earlier. The tumour was dissected free and removed, leaving a 5–10 mm. flat fragment attached to the underlying tissue. The removed tumour was weighed and fixed in formalin : the skin was closed with a row of clips.

*Intravenous injections* were made into the tail vein without anaesthesia. Approximately two grams of tumour, free from necrosis, were immersed in a balanced salt solution and subjected to prolonged mincing with scalpel blades. The turbid suspension of tiny fragments was then gently mashed in a glass cylinder with a loosely fitting plunger. The supernatant fluid contained single cells and clumps which the addition of trypan blue (Zeidman, McCutcheon and Coman, 1950) indicated were about 15 per cent viable. Absolute cell counts were not done routinely, although an effort was made to produce roughly comparable suspensions. Each rat received 0·2 ml. of the suspension. Preliminary trials showed that incorporation of grossly necrotic tumour resulted in rapid death of the animal : this was avoided by using care in the choice of tumour fragments.

All rats bearing grafted tumours were kept until dead or moribund. Their tumours were weighed, and a complete autopsy was done. Rats injected intravenously were kept for approximately seven weeks : by this time nearly all the tumours found could be seen with the naked eye. All tumours were examined histologically ; sections were also made of each lobe of all lungs, and of any other organ showing gross metastases.

Of the eight tumour sublines used, all were studied as ordinary subcutaneous grafts, repeated subtotal removals, and intravenous injections. Five of the tumours were also studied as intramuscular transplants and subcutaneous grafts with massage of the tumour (Table I.)

### RESULTS

The results from the standpoint of metastases formation are outlined in Table I. The essential features can be summarized as follows ;

1. Spontaneous metastases occurred in at least one instance from six of the eight tumour sublines studied. Ordinary subcutaneous grafts yielded metastases in three of the eight strains, and intramuscular grafts produced metastases in

TABLE I.—*Number of Rats with Metastases*

| Tumour line. | Ordinary sub-cutaneous grafts. | Intra-muscular grafts. | Sub-cutaneous massaged. | Sub-cutaneous with repeated subtotal removal. | Total with spon-taneous metastases. | Intra-venous injections. |
|---|---|---|---|---|---|---|
| 52 FM | 0/8 | — | — | 0/10 | 0/18 | 0/8 |
| 45 FM | 0/10 | — | — | 0/8 | 0/18 | 0/8 |
| 47 FM | 0/7 | — | — | 2/9 | 2/16 (12·5%) | 2/22 (9·1%) |
| 35 FM | 3/7 | 2/7 | 1/10 | 3/13 | 9/37 (24·3%) | 4/11 (36·4%) |
| 42 FM | 0/6 | 1/4 | 1/6 | 0/16 | 2/32 (6·3%) | 0/15 (0%) |
| 41 FM | 0/8 | 0/7 | 0/6 | 1/10 | 1/31 (3·2%) | 0/8 (0%) |
| 17 FM | 2/7 | 2/5 | 1/6 | 0/4 | 5/22 (22·7%) | 3/12 (25%) |
| 53 FM | 1/6 | 4/7 | 1/7 | 5/8 | 11/28 (39·3%) | 5/12 (41·7%) |
| Total | 6/34 (17·6%) | 9/30 (30%) | 4/35 (11·4%) | 9/51 (17·6%) | 28/150 (18·7%) | 12/58 (20·7%) |

four of the five strains grafted in this manner. Two sublines failed to metastasize under any condition studied. When metastases occurred, they were always present in the lungs ; a few showed metastases to other organs in addition. (Fig. 3–18).

2. Neither repeated subtotal removal nor massage of the tumour increased the overall incidence of metastases. Two sublines, however (47 FM and 41 FM) yielded spontaneous metastases only after repeated removals. (Fig. 3–10.)

3. Intravenous injection of tumour cells produced lung metastases only in the case of those tumours which were capable of spontaneous metastases. The incidence of metastases from this technique roughly paralleled, with each subline and as a whole, the incidence of spontaneous metastases. This correlation should probably be regarded as fortuitous since the amount of tumour injected was purely arbitrary, and was only roughly equal among the various strains.

TABLE II.—*Primary Tumour Weight, Life Span and Tumour Growth Rate under Varying Environmental Conditions*

| | Average weight of primary tumour in grams. | | Average life span of tumour-bearing rat in weeks. | | Average growth rate* of tumour in grams per week. | |
|---|---|---|---|---|---|---|
| | Rats without metastases. | Rats with metastases. | Rats without metastases. | Rats with metastases. | Rats without metastases. | Rats with metastases. |
| Ordinary subcutaneous grafts | 193±20·9 | 128±29·4 | 8·0±0·46 | 6·3±1·22 | 21·5±2·2 | 19·0±3·6 |
| Intramuscular grafts | 171±15·5 | 191±31·8 | 10·7±0·85 | 13·3±4·43 | 15·9±1·2 | 15·6±1·8 |
| Subcutaneous grafts —massaged | 127±11·1 | 196±42·5 | 7·0±0·69 | 11·5±2·35 | 16·5±1·1 | 16·4±1·6 |
| Subcutaneous grafts with repeated subtotal removal | 135±15·0 | 170±24·7 | 14·1±1·17 | 21·1±2·68 | 10·7±0·8 | 10·3±1·9 |
| Average of all rats | 152 | 171 | 10·2 | 14·0 | 15·5 | 14·7 |

* Calculated from growth rates of individual tumours.

In Table II an analysis of the final tumour size, life span and growth rates is given for those tumours which were studied under all four conditions, and a comparison is made between tumours which metastasized and those which remained localized. The data indicate that :

(i) Subcutaneous tumours grew more rapidly and killed the animal at an earlier date than did intramuscular grafts.

(ii) The growth rate of the tumours which metastasized was not significantly different from that of those which did not. On an average it would appear that there was slightly longer survival and a greater final tumour size where metastases occurred. The individual variations were, however, so great that the differences are not statistically significant. In fact, one of the most striking points noted throughout the experiment was, in spite of fairly uniform growth rate, the tremendous variation in individual survival time, and the lack of correlation between life span and metastasis formation.

TABLE III.—*Primary Tumour Weight, Life Span and Tumour Growth Rates of Three Tumour Sublines*

| | Average weight of primary tumour in grams. | | Average life span of tumour-bearing rat in weeks. | | Average growth rate* of tumour in grams per week. | |
|---|---|---|---|---|---|---|
| Tumour. | Rats without metastases. | Rats with metastases. | Rats without metastases. | Rats with metastases. | Rats without metastases. | Rats with metastases. |
| 35 FM | 96± 9·7 | 103±19·0 | 6·0±0·8 | 7·6±1·7 | 19·8±2·1 | 16·6±2·7 |
| 17 FM | 129±18·7 | 178±28·4 | 10·8±1·5 | 11·4±0·7 | 12·7±1·8 | 15·9±2·5 |
| 53 FM | 194±27·3 | 233±20·0 | 13 ±1·5 | 18·5±1·5 | 16·8±2·2 | 13·1±1·7 |

* Calculated from growth rates of individual tumours.

### DISCUSSION

The studies described were commenced in the belief that the tumours used would not metastasize spontaneously under the usual transplantation procedures, and were carried out in the hope of finding under what circumstances metastases could be made to occur. The demonstration that an appreciable number metastasized from simple subcutaneous and intramuscular grafts was unexpected and is difficult to reconcile with the findings of previous workers. Little importance can be ascribed to the fact that some of the tumours used have been propagated for a considerable time, since the 53 FM tumour, which yielded the highest rate of metastases, arose only a few weeks before the experiments were performed. A very careful search was made for metastases in the present study, but even a cursory examination of the lungs would have given essentially the same results as far as metastatic frequency is concerned. The experiments do establish beyond doubt that some examples of this particular type of sarcoma are true malignant neoplasms in behaviour as well as in appearance.

From the present study there is little evidence that massage or repeated subtotal ablation of a tumour affects appreciably the incidence of metastases. Possibly these effects are more readily demonstrated with tumours more prone to metastasize ; a definite trend might have been established with the present group of tumours had more animals been used. Differences in the vascularity and consistency of individual tumour types may also account for the discrepancy between the results of the present study and those of previous workers. While repeated removals failed to increase the overall incidence of metastases, two strains metastasized only after this procedure, and some evidence of increasing tumour anaplasia during the life span of the host was seen (Fig. 10). The general failure of this procedure may have been due to insufficient prolongation of life; with each succeeding removal the tumours became increasingly difficult to dissect free, and operative mortality rose after two or three removals.

The results indicate that, with this particular group of tumours, the occurrence of metastases is not dependent solely upon vascular invasion and embolization ; intravenous injections produced tumours only with those subline tumours capable of spontaneous metastasis. While this seems completely at variance with the findings of some workers (Weil, 1913 ; Andervont and Shimkin, 1941), it seems possible that different types of tumours may vary in this regard. Some tumours may principally lack the ability to invade vessels and yet be quite capable of growth when artificially disseminated, while others may be more notably deficient in their powers of forming new tumour foci from embolic origin. The failure to influence the incidence of metastases by mechanical interference or intravenous injection in the present study does not establish that factors necessary for metastasis reside solely in the tumour cells.

Although the data show no significant relation between metastasis formation and final tumour size, life span or growth rate, the extensive work of Wood and his associates (1954) shows that a large number of animals may be necessary to establish a significant trend. The present data are based on a relatively small number of animals, and the results show too much individual variation. They therefore probably should not be considered to be evidence against the conclusions of these workers.

Three characteristics of the tumours under investigation deserve consideration :

1. The time required for spontaneous metastases to develop is variable and unpredictable in spite of the fact that venous invasion is common.

---

### EXPLANATION OF PLATES

All sections were stained with Haematoxylin and eosin.

FIG. 1.—52 FM tumour showing fibrous structure. No metastases occurred from this subline. × 80.
FIG. 2.—45 FM tumour showing immature fat cells. No metastases were observed. × 80.
FIG. 3.—47 FM tumour showing fibrous tissue and early bone formation. Spontaneous metastases occurred after repeated subtotal removal. × 80.
FIG. 4, 5, 6, 7 and 8.—Spontaneous metastases from 47 FM tumour after repeated subtotal removal. Fig. 4, 5 and 6 show pulmonary metastasis composed of fibrous tissue and bone. Fig. 6 (from a decalcified section) demonstrates the extreme degree of bone formation encountered in some pulmonary nodules. Metastasis to the kidney is seen in Fig. 7, and to the myocardium (left ventricle) in Fig. 8. Fig. 4 × 50. Fig. 5, 6, 7 and 8. × 80.
FIG. 9.—41 FM tumour showing early venous invasion. This tumour metastasized only after repeated subtotal removal. × 80.
FIG. 10.—Spontaneous lung metastasis from 41 FM tumour. The cells appear more pleomorphic and anaplastic than those seen in the primary tumours. × 80.
FIG. 11.—42 FM tumour. × 80.
FIG. 12.—Spontaneous lung metastasis from 42 FM tumour. × 80.
FIG. 13.—35 FM tumour. × 80.
FIG. 14.—Spontaneous lung metastasis from 35 FM tumour. × 80.
FIG. 15.—17 FM tumour. × 80.
FIG. 16.—Spontaneous lung metastasis from 17 FM tumour. × 80.
FIG. 17.—53 FM tumour. × 80.
FIG. 18.—Spontaneous lung metastasis from 53 FM tumour. × 50.

1

2

3

4

5

6

Wallace.

7

8

9

10

11

12

13

14

15

16

17

18

Wallace.

2. Intravenous inoculation is not uniformly successful in producing lung tumours.

3. A large amount of tissue is apparently needed for transplantation of the tumours.

It has been customary to transplant this tumour by means of relatively large fragments, and unpublished trials by myself indicate that very large numbers of cells are, in fact, required for successful takes. All three of these characteristics might be explained on the basis of a diverse cell population within the tumour. Some very malignant tumours, such as ascites tumours and lymphosarcomas, are transplantable with only a single cell (Furth and Kahn, 1937 ; Hauschka, 1953). It seems obvious that such tumours must be composed almost entirely of transplantable cells, each capable of independent growth. In contrast, tumours such as those used in the present study may contain only a small proportion of such cells. Thus, uniform success in transplantation might require a large amount of tissue, and, of all the cells gaining entrance into the circulation, either spontaneously or artificially, only occasional clumps might be successful in producing metastases. The unpredictable behaviour of some human tumours might have the same explanation. Death of tumour emboli in small vessels has been observed by innumerable workers with both human and experimental tumours ; likewise, the concept of a heterogeneous tumour cell population is not an original one. Nonetheless, lack of uniformity of transplantability within a given tumour population might be investigated more extensively as an explanation of why some tumours fail to show a consistent pattern of spread and produce occasional solitary metastases in spite of obvious venous invasion. The proportion of cells capable of independent growth may be the determining factor in such behaviour.

### SUMMARY

A group of sarcomata arising from a single transplantable rat fibroadenoma has been studied under a variety of conditions in order to determine under what circumstances metastases would occur. Contrary to the reports of previous workers, an appreciable number of these tumours are capable of spontaneous metastasis and should be considered true malignant tumours. Massage of the transplanted tumour did not increase the frequency of metastases. While repeated subtotal removal did not increase the overall incidence of metastases, it produced spontaneous metastases in two sublines which did not occur under any other circumstances. Artificial metastasis by intravenous inoculation was successful only with those sublines which were capable of spontaneous metastasis. While the growth rate of the tumours was fairly uniform, there was great variation in the survival time, final tumour size, and time required to produce metastases. Possible explanations for some of the results have been discussed.

This work was supported by a grant-in-aid from the National Cancer Institute of Canada.

I am grateful to Mrs. Susan Davis, Mrs. Grace Strickland and Miss Bette Byrns for technical assistance. My thanks are due to Miss Lizetta Nason for taking the photographs and to Dr. Margaret Owchar of the Manitoba Cancer Institute for evaluating the statistical data.

### REFERENCES

AGOSIN, M., CHRISTEN, R., BADINEZ, O., GASIC, G., NEGHME, A., PIZZARO, O. AND JARPA, A.—(1952) *Proc. Soc. exp. Biol. N.Y.*, **80**, 128.
ANDERVONT, H. B. AND SHIMKIN, M. B.—(1941) *J. nat. Cancer Inst.*, **2**, 151.
BASERGA, R. AND SHUBIK, P.—(1954) *Cancer Res.*, **14**, 12.
*Idem* AND BAUM, J.—(1955) *Ibid.*, **15**, 52.
BONNE, C.—(1925) *C. R. Soc. Biol. Paris*, **93**, 312.
CIRIO, L. AND BALESTRA, G.—(1930) *Pathologica*, **22**, 451.
CLUNET, J.—(1910) 'Recherches Experimentales sur les Tumeurs Malignes', Paris (Steinheil) (Quoted in Oberling, C.—(1952) 'The Riddle of Cancer'. New Haven (Yale University Press)).
CRABB, E. D.—(1949) *J. nat. Cancer Inst.*, **10**, 581.
DUNHAM, L. J. AND STEWART, H. L.—(1953) *Ibid.*, **13**, 1299.
EMGE, L. A.—(1938) *Arch. Path.*, **26**, 429.
ENGELL, H. C.—(1955) *Acta chir. scand.*, Supplement 201.
FURTH, J. AND KAHN, M. C.—(1937) *Amer. J. Cancer*, **31**, 276.
GAYLORD, H. R. AND SIMPSON, B. T.—(1916) *J. Cancer Res.*, **1**, 379.
GOLDIE, H., JEFFRIES, B. R., JONES, A. M. AND WALKER, M. (1953) *Cancer Res.*, **13**, 566.
*Idem*, WALKER, M., JEFFRIES, B. R. AND GUY, R.—(1955) *Proc. Amer. Ass. Cancer Res.*, **2**, 19.
HAUSCHKA, T. S.—(1953) *Ibid.*, **1**, 24.
HEIMAN, J.—(1934) *Amer. J. Cancer*, **22**, 497.
KALISS, N., BORGES, P. R. F. AND DAY, E. D.—(1954) *Cancer Res.*, **14**, 210.
KNOX, L. C.—(1921) *J. Cancer Res.*, **6**, 192.
LOEB, L. AND FLEISHER, M. S.—(1916) *Ibid.*, **1**, 427.
MARSH, M. C.—(1927) *Ibid.*, **11**, 101.
MARTINEZ, C. AND BITTNER, J. J.—(1955) *Proc. Soc. exp. Biol. N.Y.*, **89**, 569.
*Idem*, MIROFF, G. AND BITTNER, J. J.—(1956) *Cancer Res.*, **16**, 313.
MAZZACUVA, G.—(1933) *Pathologica*, **25**, 856.
MILLAR, M. J. AND NOBLE, R. L.—(1954*a*) *Brit. J. Cancer*, **8**, 485.—(1954*b*) *Ibid.*, **8**, 508.
POMEROY, T. C.—(1954) *Cancer Res.*, **14**, 201.
ROUSSY, G., OBERLING, C. AND GUERIN, M.—(1936) *Bull. Ass. franç. Cancer*, **25**, 592. (Abstracts in *Amer. J. Cancer*, (1937), **30**, 147.
SELBIE, F. R.—(1941) *Brit. J. exp. Path.*, **22**, 156.—(1942) *Ibid.*, **23**, 61.
TADENUMA, K.—(1923) *Z. Krebsforsch.*, **20**, 394.
*Idem* AND OKONOGI, S.—(1924) *Ibid.*, **21**, 168.
TAKAHASHI, M.—(1915) *J. Path. Bact.*, **20**, 1.
TANNENBAUM, A. AND SILVERSTONE, H.—(1952) *Cancer Res.*, **12**, 302.
TERRANOVA, T. AND CHIOSSONE, F.—(1952) *Boll. Soc. Biol. sper.*, **28**, 1224.
TYZZER, E. E.—(1913) *J. med. Res.*, **28**, 309.
VON ESSEN, C. F. AND KAPLAN, H. S.—(1952) *J. nat. Cancer Inst.*, **12**, 883.
WATANABE, S.—(1954) *Cancer*, **7**, 215.
WEIL, R.—(1913) *J. med. Res.*, **28**, 497.
WOOD, J. S., HOLYOKE, E. D., CLASON, W. P. C., SOMMERS, S. C. AND WARREN, S.—(1954) *Cancer*, **7**, 437.
*Idem*, HOLYOKE, E. D., SOMMERS, S. C. AND WARREN, S.—(1955) *Johns Hopk. Hosp. Bull.*, **96**, 93.
*Idem*, HOLYOKE, E. D. AND YARDLEY, J. H.—(1956) *Proc. Amer. Ass. Cancer Res.*, **2**, 157.
ZEIDMAN, I., MCCUTCHEON, M. AND COMAN, D. R.—(1950) *Cancer Res.*, **10**, 357.